# Arrhythmia related to hypertensive left ventricular hypertrophy in Iraqi patients: frequency and outcome

**DOI:** 10.25122/jml-2022-0214

**Published:** 2022-09

**Authors:** Ameen Abdulhasan Al Alwany

**Affiliations:** 1 College of Medicine, University of Baghdad, Baghdad, Iraq

**Keywords:** arrhythmia, hypertension, left ventricular mass index, atrial fibrillation

## Abstract

Left ventricular hypertrophy (LVH) caused by high blood pressure is linked to increased mortality and arrhythmia risk. This study aimed to evaluate arrhythmia in hypertensive patients due to left ventricular hypertrophy (LVH). A cross-sectional study was performed, assessing participants' blood pressure, echocardiography and electrocardiography, and Holter monitoring in certain cases. There were 300 hypertensive patients >18 years attending the cardiology unit of Baghdad medical city. The study was conducted between January–June 2022. The electrocardiograms at rest for 300 adults with hypertension were investigated. 130 (43.5%) were females, and 170 (56.5%) were males. The mean age of participants was 58 years. Forty-nine (16.3%) patients had arrhythmia. As compared to those without arrhythmia, participants with arrhythmia were older (62.3 *vs*. 56.1, p=0.03), had a greater prevalence of left ventricular hypertrophy (24.5% *vs*. 12.7%, p=0.026), and more prone to experience cardiac failure (32.7% *vs*. 8.5%, p<0.011). Atrial fibrillation was found in 6 (27.2%) female patients and 5 (18.5%) males. In addition, two (7.4%) male patients and one female patient (4.5%) had atrial flutter, and premature ventricular contractions (PVCs) were noted in 10 (37%) and 11 (50%) patients. Left ventricular mass index (LVMI) was 103 gm/m^2^ in female patients and 119.2 gm/m^2^ in males. Palpitation was present in 22 (44.9%) female patients and 27 (55.1%) males. The study revealed that hypertensives with LVH have an arrhythmia frequency of 16.3%. The most common arrhythmias were atrial fibrillation and premature ventricular complex.

## INTRODUCTION

Arterial hypertension is a substantial risk factor for unfavorable cardiovascular events globally due to its link to atherosclerosis, peripheral artery disease, coronary artery disease (CAD), chronic renal disease, heart failure, and stroke [1]. Additionally, numerous epidemiological researchers noted the connection between hypertension and various cardiac arrhythmias, which has significant ramifications for the morbidity and even mortality of hypertensive patients [2]. The hypertensive cardiac disease may be present when atrial fibrillation (AF) first appears. Detection of AF in hypertension patients may be a sign of hypertensive organ damage [3].

Further contributing to the advancement of AF is uncontrolled hypertension. The risks of systemic embolism and stroke, tachycardia-induced cardiomyopathy, chronic heart failure (HF), and acute HF exacerbations are all raised in hypertensive patients with AF [4, 5]. Additionally, patients with hypertension might also have an underlying condition that is connected to AF episodes. On the other hand, AF sufferers should check their blood pressure frequently due to their frequent co-existence. Early detection of potential hypertension is essential to begin appropriate medical therapy and lower the risk of thrombosis and bleeding. Diastolic blood pressure measurement may be challenging in individuals with persistent atrial fibrillation, particularly with equipment used at home, although systolic blood pressure measurements can be accurate [4, 5].

There are still no clear indications for using echocardiography to treat hypertension [5]. Transthoracic echocardiography (TTE) is the most commonly applied method. This gives the physician access to real-time measurements of the heart's dimensions, composition, and functionality throughout the cardiac cycle. Stress echocardiography is another important and practical application of these techniques [5]. Stress echocardiography is the combination of either pharmacological or physical stress on the cardiac structures and traditional transthoracic echocardiography to evaluate aberrations in wall motion. Running on a treadmill is one form of physical stress, and taking some drugs can also cause stress [6].

The most popular non-invasive technique for examining cardiac anatomy is echocardiography. Thin cross-sections of cardiac structures, such as the left and right atriums, left and right ventricles, valves, and related valvular structures can be obtained using echocardiography [5, 7].

Left atrial enlargement is brought on by the sluggish growth of hypertension. Large LA creates an ideal environment for a sinus rhythm to loop inside itself, resulting in AF. Atrial contraction is becoming increasingly important for properly filling the left ventricle (LV). This filling is offset by the sudden start of AF, which results in inadequate LV filling and increased LA pressure that travels to the pulmonary capillaries. The fibrotic region can serve as a substrate for developing ectopic rhythms, which causes chronic intractable AF and increases the risk of clot formation and systemic embolism [8].

A smaller LV cavity and a decreased stroke volume are the results of excessive left ventricular hypertrophy (LVH). Additionally, it causes the distinct levels of the LV contraction to become disorganized. Strain imaging can be used to measure this. When LVH is too high, different regions experience maximum contractions at various times. A strain image can be used to evaluate this mechanical dispersion [9]. There is growing evidence that ventricular arrhythmias and abrupt cardiac death can result from dispersion above the standard deviation (SCD). In the future, strain imaging echo technologies may make it possible to predict which subsets of these patients are most prone to develop ventricular fibrillation and SCD [8, 9].

## MATERIAL AND METHODS

A cross-sectional study was conducted, including adult participants with hypertension aged 18 years and above who attended the Cardiology Unit of the Baghdad medical city between January–June 2022. Blood pressure, electrocardiography, and echocardiography were assessed for all participants. In addition, Holter monitoring was performed in certain cases.

Age and sex, blood pressure, medicines, and clinical diagnosis, were all listed on the ECG request and sheet. Prognostic indicators for hypertension are left ventricular mass, left atrial function, systolic function, diastolic activity, and size. Three-dimensional echo, tissue Doppler, and strain imaging are more recent echo techniques used in evaluating hypertension patients in addition to standard echo methods [5]. Blood pressure evaluation and hypertension classification after participants had been seated for at least five minutes, systolic (SBP) and diastolic (DBP) blood pressures were assessed on the left arm using a standardized methodology. The blood pressure reading was accurate within 2 mmHg.

Hypertension was diagnosed as SBP ≥140 mmHg and/or DBP ≥90 mmHg or treated with antihypertensive drugs. Electrocardiogram standard supine resting 12-lead ECG was recorded using a machine complying with the recommendations of the American Heart Association for technical specifications. The ECG report was contained in the ECG request form for each patient.

These records were screened for the following abnormal rhythms: atrial fibrillation, atrial flutter, atrial and ventricular premature complexes, and supraventricular and ventricular tachyarrhythmias. Those with arrhythmias were confirmed and coded by a cardiologist. The criteria for arrhythmias were based on the standard ECG criteria, and Holter monitoring showed significant premature ventricular contractions.

## RESULTS

The ECG at rest for 300 Iraqi adults with hypertension was studied. There were 170 (56.5%) males and 130 (43.5%) females with a male to female ratio of 1.3:1. The mean age of the patients was 58 years. The mean ages of patients with and without arrhythmia were 62 and 56, respectively (p=0.010) (Table 1).

**Table 1:** Comparison of data between two groups.

	With arrhythmia (n=49)	Without arrhythmia (n=251)	P-value
	Frequency %	Frequency %	
Mean age	62.3±	56.1±	0.010
Sex			
**Male (no.)**	26	142	0.750
**Female (no.)**	23	109	0.670
SBP (mmHg)	180	165	0.001
DBP (mmHg)	95	85	0.039
HF	14	22.5	0.011
ECG LVH	12	38.5	0.026

The frequency of arrhythmias increased with age and was more prevalent in patients 60 years and older (Table 2). Forty-nine (16.3%) patients had arrhythmia, more among females than males (17% *vs*. 15%, p=0.490) though it was not significant.

**Table 2:** Distribution of age among patients with arrhythmia.

Age (years)	Frequency (n) (%)
18–29	2 (4)
30–39	3 (6.1)
40–49	5 (10.2)
50–59	7 (14.3)
60–69	15 (30.6)
≥70	17 (34.6)

AF was more common in females than males (4.63% *vs*. 3.2%, p=0.840), although not statistically significant. As compared to those without arrhythmia, patients with arrhythmia were older (62 *vs*. 56, p<0.030), had a higher SBP (180±20 *vs*. 165±20, p<0.0001) and DBP (95±10 *vs*. 85±10, p=0.039) and LVH (24.5% *vs*. 12.7%, p=0.026), and more likely to be in hypertension (14% *vs*. 22.5%, p<0.001) (Table 3). The patterns and proportions of arrhythmia are shown in Table 3. The most frequent arrhythmias were PVC and AF, representing 42.9% and 23.5%, respectively. Table 4 describes the relations between the age of patients and the pattern of arrhythmias.

**Table 3:** Proportions and patterns of arrhythmia.

	Female (n=22)	Male (n=27)	Total (n=49)	P-value
	Frequency %	Frequency %	Frequency %	
PAC	3 (13.6%)	6 (22.2%)	9 (18.3%)	0.010
PVC	11 (50%)	10 (37%)	21 (42.8%)	0.018
AF	6 (27.2%)	5 (18.5%)	11 (22.4%)	0.022
AFL	1 (4.5%)	2 (7.4%)	3 (6.1%)	0.437
AT	1 (4.5%)	2 (7.4%)	3 (6.1)	0.460
VT	-	2 (7.4%)	2 (4%)	-
VF	-	-	-	-

PAC – Premature atrial complexes; PVC – Premature ventricular complexes; AF – Atrial fibrillation; AFL – Atrial flutter; AT – Atrial tachycardia; VT – Ventricular tachycardia; VF – Ventricular fibrillation.

**Table 4:** Frequency of arrhythmia in relation to age.

	PAC	PVC	AF	AFL	AT	VT	VF
	n=9	n=21	n=11	n=3	n=3	n=2	
18–29	-	1	-	-	1	-	-
30–39	1	1	-	-	1	-	-
40–49	1	2	-	1	1	-	-
50–59	2	3	2	-	-	-	-
60–69	3	6	4	2	-	-	-
<70	2	8	5	-	-	-	-

The study also revealed that arrhythmia was more common in older patients, as 64% occurred in those 60 years and above.

## DISCUSSION

The study showed that PVC and AF were the most common arrhythmias in adult patients with hypertension in our setting, constituting 42.8% and 22.4%, respectively. This is consistent with the findings in some previous studies [9]. PVC is a common problem in clinical practice [10].

The mechanism of the arrhythmia may be automatic, activity-triggered, or re-entry, and increased sympathetic tone and QTc prolongation may play a role in the progression to ventricular tachyarrhythmia, particularly in the presence of LVH and ventricular dysfunction [11]. The study also revealed that arrhythmia was more common in older patients, as 65.2% occurred in those 60 years and above. This finding also corroborates reports from other studies [12, 13].

The increased prevalence of arrhythmia and other ECG findings in the elderly is due to the increased prevalence of cardiovascular disease and the impact of physiological aging changes. Electrocardiographic changes may occur due to the substantial changes in cardiovascular structure and function linked with aging [14]. Thus, aging might be an important factor in abnormal findings and the appearance of arrhythmias in conventional surface 12-lead ECG [15].

In our study, although the overall prevalence of AF was 3.8%, 4.3%, 43.5%, and 52.2%, the cases occurred in patients <50 years, 50–69 years, and 70 years and older, respectively. Atrial fibrillation prevalence was significantly correlated with age. This is similar to findings reported by Alan et al. [16] and other authors [17].

AF is one of the most common arrhythmias in elderly persons and a significant risk factor for ischemic stroke, increasing the chance of having one and contributing to fifteen percent of all strokes in the US. In addition to lowering the quality of life and heart performance, symptomatic AF is linked to higher medical expenses and mortality risk [18].

In our study, although not statistically significant, females were more likely to have an arrhythmia than males, including AF, except for those at and above 70 years. This corresponds to the study by Yamaguchi et al. [19], which showed that PAC, PVC, and AF were more frequent in men than women, but only in the older age stratum [20].

This study also showed that hypertensive patients with arrhythmia were significantly more likely to be in heart failure (HF) than those without HF. There is a two-way relationship between arrhythmia and HF [20]. While all kinds of HF bear a heavy load from arrhythmia, and some even perpetuate it, structural substrates for arrhythmia are common in HF, whatever the underlying reason, including myocardial hypertrophy, myocardial fibrosis, and ventricular dilatation. In HF at the cellular level, myocytes may be exposed to rising stretch and wall tension, excessive ischemia, catecholamines, and electrolyte imbalance [5, 21]. Arrhythmogenic sudden cardiac death is more common in HF patients due to the complicated interaction of these variables. In our study, patients with arrhythmia also had significantly higher SBP and DBP and were more likely to have LVH. While increased SBP creates more wall strain, whereas rises in DBP promote the remodeling of the ventricle, an increase in myocardial oxygen demand, myocardial ischemia, and eventually the advancement of the heart's maladaptive processes that result in decompensated HF and/or breakdown of normal conduction patterns with increased propensity for abnormal automaticity or activation of reentrant pathways in the myocardium which may generate arrhythmia [22]. In the study, arrhythmias were more likely found in older HF patients with higher blood pressure and LVH [22, 23].

## CONCLUSIONS

In patients with hypertension, left ventricular hypertrophy raises the chance of ventricular and atrial arrhythmias, systolic and diastolic heart failure, and sudden cardiac arrest. Regression of LVH and reduction in cardiovascular morbidity and death are made possible by controlling arterial blood pressure.
